# Prevalence of *Anaplasma phagocytophilum *infection and effect on lamb growth

**DOI:** 10.1186/1751-0147-53-30

**Published:** 2011-05-13

**Authors:** Lise Grøva, Ingrid Olesen, Håvard Steinshamn, Snorre Stuen

**Affiliations:** 1 Norwegian Institute for Agricultural and Environmental Research (Bioforsk), Organic Food and Farming Division, Gunnars veg 6, 6630 Tingvoll, Norway; 2 Department of Animal and Aquacultural Science, Norwegian University of Life Sciences, Postbox 5003, 1432 Ås, Norway; 3 Nofima Marin, Postboks 5010, 1432 Ås, Norway; 4 Section for small ruminant research, Norwegian School of Veterinary Science, 4325 Sandnes, Norway

## Abstract

**Background:**

A major challenge in sheep farming during the grazing season along the coast of south-western Norway is tick-borne fever (TBF) caused by the bacteria *Anaplasma phagocytophilum *that is transmitted by the tick *Ixodes ricinus*.

**Methods:**

A study was carried out in 2007 and 2008 to examine the prevalence of *A. phagocytophilum *infection and effect on weaning weight in lambs. The study included 1208 lambs from farms in Sunndal Ram Circle in Møre and Romsdal County in Mid-Norway, where ticks are frequently observed. All lambs were blood sampled and serum was analyzed by an indirect fluorescent antibody assay (IFA) to determine an antibody status (positive or negative) to *A. phagocytophilum *infection. Weight and weight gain and possible effect of infection were analyzed using ANOVA and the MIXED procedure in SAS.

**Results:**

The overall prevalence of infection with *A. phagocytophilum *was 55%. A lower weaning weight of 3% (1.34 kg, p < 0.01) was estimated in lambs seropositive to an *A. phagocytophilum *infection compared to seronegative lambs at an average age of 137 days.

**Conclusions:**

The results show that *A. phagocytophilum *infection has an effect on lamb weight gain. The study also support previous findings that *A. phagocytophilum *infection is widespread in areas where ticks are prevalent, even in flocks treated prophylactic with acaricides.

## Background

Tick-borne fever (TBF) is one of the main challenges in Norwegian sheep farming during the grazing season [1]. TBF is caused by the bacteria *Anaplasma phagocytophilum*, transmitted by the tick *Ixodes ricinus*, and may cause direct (lamb deaths) and indirect loss (reduced growth) in sheep farming. The normal distribution area of *I. ricinus *ticks in Norway is the coastal areas of Norway as far north as Brønnøysund in Nordland county (N 65°30'), Norway [2-4]. *A. phagocytophilum *infected lambs are commonly found in areas with ticks [2,5]. Climate change (i.e. warmer winter climate), changes in land use (i.e. bush encroachment) and an increase in the deer population are factors expected to increase the populations of ticks. An extension of the northern margin of the population distribution of *I.ricinus *and to higher altitudes has been observed [6,7], and has given rise to concerns that challenges with TBF will increase in Norway in the coming years.

The main consequence of an *A. phagocytophilum *infection in sheep is the ensuing immunosuppression that may lead to secondary infections and cause both direct and indirect losses. Direct losses of ca 30% lamb mortality in a flock due to *A. phagocytophilum *infection have been observed [8,9]. The exact causes of deaths of lambs on pasture have however seldom been determined, because most lambs have been grazing on free range forest and mountain pastures with only weekly attention. Hence only a few lost lambs have been found [10-12]. The extent of indirect production loss due to TBF was 3.8 kg body weight per lamb in a study of a flock with 50 lambs [13] and experimental infection with *A. phagocytophilum *has shown to affect weight for several months after the primary infection [14]. It is also shown that prophylactic use of long-acting tetracycline against *A. phagocytophilum *has improved weight gain in lambs on pasture [15].

Several genetic variants of *A. phagocytophilum *are observed and it is shown that these cause different clinical signs with varying haematological and serological response; i.e. differences in duration of fever, maximum temperature, level of antibody titre, and weight reduction [16-18].

There is great concern about indirect and direct losses to TBF among sheep farmers in areas where *I.ricinus *is abundant. The objective of the present work was to examine the prevalence of TBF in lambs on tick-infested pastures, and to quantify the extent of weight loss of lambs that can be expected on tick-infested pastures.

## Methods

### Study population

Lambs from Sunndal Ram Circle [19] in the county of Møre and Romsdal (Mid Norway) were selected for this study (62°N, 9°E). Sunndal Ram Circle is a ram circle for the Norwegian White Sheep breed and consisted of 21 sheep farmers in 2007 and 2008 who cooperated with progeny testing of 28 ram lambs (868 matings) and elite matings by mating with a total of 280 ewes in 2007 [20]. The studied population of lambs were presumed to be grazing in tick-infested areas as *A. phagocytophilum *infection was confirmed on six farms in Sunndal Ram Circle in 2006.

The study sample included lambs from 12 of the farms in Sunndal Ram Circle that were turned out onto pasture together with their mothers in 2007 and 2008 with spring and weaning weight recordings. Data on spring and weaning weight, age at weighing, sex, rearing rank and mother were collected and obtained through The Norwegian National Sheep Recording Scheme [21]. Table 1 shows mean lamb weights and SD of the sampled lambs in 2007 and 2008. Information on lamb losses on summer pasture was collected from recordings done by the by the Norwegian Forest and Landscape Institute [22]. Cause of direct lamb losses was not determined in this study. Blood samples were collected in 2007 (n = 968) and 2008 (n = 240) during the event of collection and weighing of lambs at the farms in autumn prior to slaughter or selection for breeding. Weight scales were calibrated on the actual day of weighing.

**Table 1:** Mean (SD) of weight parameters of the study population, and county and national average.

	Study population		**Møre & Romsdal**^**1**^		**Norway **^**1**^	
	2007 (n = 968)	2008 (n = 240)	2007	2008	2007	2008
Age at weaning weight (days)	137 (9.7)	139 (7.8)	145	145	145	145
Weaning weight (kg)	45.7 (8.2)	47.6 (7.7)	42.3	44.6	44.5	45.5
Weight gain spring-weaning (g/day)	285 (54.3)	296 (59.5)	237	260	255	262

^1 ^[21,39]

### Farm characteristics and management

A questionnaire was sent to the 12 selected farmers in Sunndal ram circle to gather information on farm characteristics and management. Information on prophylactic treatment of sheep against ticks and farmers' perception of having ticks on their pastures (yes/no) is presented in table 2. The altitude in meters above sea level (masl) of the spring pastures was 0-200 (masl) for ten of the twelve farms. The remaining two farms; farm D and I, had spring pastures at 100-400 and 700 masl respectively. Altitude of summer pastures varied between 150 and 1300 masl. Spring and autumn pastures were cultivated pastures with bush vegetation. Summer pastures were mountain and valley range land with variable degree of bush and forest vegetation. Considerable between and within farm variation in bush vegetation is typical. Dominant bush vegetation species were not mapped in this study. The production system was in general similar on all farms; lambs were born indoors and then they were let onto spring pasture at the age of 0 - 4 weeks, and lambs were let onto summer pastures after a short period of grazing on spring pasture. During the autumn, lambs were gathered from summer pastures and kept on pastures close to the farm for a short period before slaughter. All sheep and lambs were treated with anthelmintics before they were let onto summer pastures. Prophylactic treatment against ticks was conducted in spring on 9 out of 12 flocks using Coopersect^® ^vet 1-2 times before lambs were let on to summer pastures. Prophylactic treatment against ticks was not conducted on three of the farms (farm B, F, I).

**Table 2:** Prevalence of seropositive lambs, weaning weight, altitude of pastures, and lamb loss per farm and year.

Farm	Number of samples 2007(2008) n		Prevalence of seropositive lambs %		Minimum altitude of pastures masl	Average weaning weight kg		Lamb loss %	
	**2007**	**2008**	**2007**	**2008**		**2007**	**2008**	**2007**	**2008**

A	30		73		0	47.9		5	
B^2^	122	79	67	14	200	44.3	48.7	8	6
C	86		78		0	48.6		0	
D	44		2		100	48.0		36 ^3^	
E	72		0		100	47.6		4	
F^1 2^	49		96		0	46.9		17	
G	173	71	58	65	0	44.8	48.2	11 ^3^	25 ^3^
H	88	90	90	81	0	43.0	46.1	17	11
I^1 2^	123		10		600	41.5		9	
J	58		55		50	50.5		1	
K	101		84		175	46.8		12	
L	22		36		150	50.0		7	

All	968	240	55	54		45.7	47.6		

^1 ^The farmer perceived that there were no ticks on their pastures

^2 ^No prophylactic treatment against ticks

^3 ^There were documented loss to wolverine (*Gulo gulo) *on these farms

### Serology

Blood samples were collected during autumn at an average age (± SD) of 137 ± 8 days. Blood samples were centrifuged for 10 min at 3200 ppm within 24 hours of sampling. Serum was extracted, frozen and later analysed by an indirect fluorescent antibody assay (IFA) to determine the antibody titre to a heterologous horse variant of *A. phagocytophilum *(formerly *Ehrlichia equi*) [5,23]. No antigen from a sheep variant of *A. phagocytophilum *was available. Briefly, a two-fold dilution of sera was added to slides pre-coated with *E.equi *antigen (Protatek International and Organon Teknika). Bound antibodies were visualized by flourescein-isothiocyanate (FITC)-conjugated rabbit-anti-sheep immunoglobulin (Cappel, Oranon Teknika). Sera were screened for antibodies at dilution 1:40, and a titre of 1:40 and higher was regarded as positive whereas titres below 1:40 were regarded as negative [5].

### Statistical analysis

#### Flock performance

Possible effects of prophylactic treatment, farmer's perception of ticks on pastures and masl of pastures (as regression effect) on the flock's prevalence of infection, direct losses on summer pasture and weaning weight was analyzed using the General Linear Model method of the GLM procedure in SAS [24]. The effect of prevalence of infection on direct loss was also estimated. The initial statistical model included all explanatory effects listed above according to the degrees of freedom available, before non-significant effects were removed by a stepwise procedure. Neither prophylactic treatment nor farmer's perception of ticks on pastures were included in the final regression model as their effect was not significant in this limited dataset. The final regression model used was:

Where Y1 is the prevalence of infection on the farm i (i = 1-12), *B*_*0 *_is the intercept, *B*_*i *_is the regression effect on masl of farm pastures i (x = 0-600) and *e*_*i *_is the random residual error.

#### Individual lamb performance

Individual lamb data on weight were analyzed using the Restricted Maximum Likelihood method of the MIXED procedure in SAS [24]. Initial statistical model included the effects of age at weighing (as regression effect), serology, age of mother, sex and rearing rank as fixed effects and farm, year, father and mother as random effects. The final models used were:

Where Y2 is the weaning weight and Y3 is the weight gain on summerpasture (spring to weaning) of the individual q (q = 1-1208); μ is the overall mean, A is the regression of the fixed effect of age at recording of weaning weight (days); Ser is the fixed effect of the serology result (i = 0, 1; where 0 = seronegative to *A. phagocytophilum *and 1 = seropositive to *A.phagocytophilum*); AM is the fixed effect of age-group of mother (j = 1, 2, 3, 4; where age group 1 = one year old, 2 = two year old, 3 = three year old, 4 = four years and older); S is the fixed effect of sex (k = 1, 2; where = male and 2 = female); R is the fixed effect of rearing rank (l = 11, 21, 22, 31, 32, 33, 41, 42, 43, 44; where the first digit is birth rank and the second digit is rank when let on to pasture); f*y is the random effect of farm-year (m = 2007, 2008) (n = 1 - 12); m is the random effect of mother (o = 1-618); e is the random residual error. All interactions with fixed effects were included in the initial analyses, but were removed subsequently if they did not show significant effect on weaning weight. Heterogeneous variance for male and female lambs was taken into account.

An analysis of variance for the explanatory effects on weaning weight was done using the GLM procedure in SAS [24].

## Results

### Serology and farm characteristics

Infection with *A. phagocytophilum *was widespread in Sunndal Ram Circle (Table 2). Positive samples were shown on 11 of the 12 farms and the proportion of antibody positive samples on these farms varied between 2 and 96%. On eight farms, 55% or more of the samples were antibody positive. Overall, 55% of the samples were positive for antibodies to *A. phagocytophilum*.

Prophylactic treatment against ticks was not conducted on three of the farms (farm B, F, I) of which two (farms F, I) perceived that there were no ticks on their pastures. On farm E no seropositive lambs were observed, but the farmer perceived that there were ticks on the pastures and used prophylactic treatment. Seroprevalence on farm F and I was 96% and 10%, respectively, and on farm I all pastures were above 600 masl. Infected lambs with *A. phagocytophilum *were observed on farms in spite of prophylactic treatment against ticks, farmers' perception of no ticks on pasture and high altitude of pasturing. The statistical model 1, however, showed that masl had a significant (p = 0.038) effect on prevalence of *A. phagocytophilum *(Table 3). There was no significant effect of prophylactic treatment and farmer's perception on prevalence of infection, lamb loss and weaning weight.

**Table 3:** Results for the analysis of variance on weaning weight of lambs.

Effect	Degrees of freedom	Marginal sum of squares	**Marginal increase in R**^**2 **^**× 100**
Mother (farm)	560	25210.22	31.57***
Sex	1	2202.21	2.76***
Rearing rank	8	1135.34	1.42***
Rearing rank (farm year)	21	840.89	1.05
Sex (farm year)	14	839.12	1.05**
Age at recording of weaning weight	1	520,70	0.65***
Age of mother	3	315.92	0.40*
Antibody result	1	264.19	0.33**
Farm (year)	3	168.90	0.21

Error	538	11679.95	-
Model	669	68174.59	85.37

Level of significance different from zero for Marginal SS (type III SS) ***p < 0.0001 **p < 0.001 *p < 0.01

### Production loss

The analysis of variance for weaning weight presented in Table 3 shows that effect of the mother explained most variation of weaning weight (32.6%). Here, both additive genetic and maternal effects are included. Antibody results only explained a small but significant proportion of the variance of weaning weight (0.3%).

There was a significant difference (± SE) between Least Square Means (LSM) of antibody positive and antibody negative lambs of 1.34 ± 0.412 kg weaning weight (p < 0.01) and 10.4 ± 3.3 g daily weight gain (p < 0.01) (Table 4). The weight difference amounts to 3% of average weaning live weight of lambs in Norway. There was no significant difference of spring weight between antibody positive and antibody negative lambs.

**Table 4:** Least Square Means of weight recordings of lambs, with S.E. and p-value of the LSM difference.

	Antibody negative	Antibody positive	LSM difference	**s.e**.	p-value
Weaning weight (kg)	45.10	43.77	1.34	0.412	0.0012*
Spring body^1 ^weight (kg)	13.87	13.74	0.14	0.162	0.4045
Daily weight gain summer pastures^2 ^(g/day)	278.4	268.0	10.4	3.31	0.0018*

* Statistically significant

^1 ^Spring body weight: Age at spring body weight is used in the model. 18 observations are not used due to missing values.

^2 ^Daily weight gain summer: 18 observations are not used due to missing values.

Lamb direct loss during the summer grazing period on the 12 farms varied from 0 to 36%. Predators caused lamb losses in these grazing areas, and lamb losses to wolverine (*Gulo gulo*) were documented in two flocks (Table 2). Losses on farms with no documented losses to predators, varied between 0 - 17%, and four of the farms had losses above country average in 2007. The actual causes of deaths in general were unknown in this study, which is the general case for most lamb losses during summer pasturing [25,12].

## Discussion

### Prevalence

The overall seroprevalence of *A.phagocytophilum *of 55% among lambs in this study is lower than earlier observations of 80% seroprevalence of lambs grazing on *I.ricinus *infested pastures [5]. It is indicated in a UK study that probably 100% of lambs grazing on tick-infested pastures will acquire *A. phagocytophilum *infection [26]. Some of the flocks in the present study were, however, grazing in mountain range land with presumably low tick density [3]. This may explain the relatively lower seroprevalence of *A. phagocytophilum *on some farms. On one farm (farm M), all sheep were grazing at 600 masl and higher, where ticks earlier have not commonly been found in Norway [3]. On this farm 10% (n = 12) of the lambs were seropositive. Our finding that prevalence of *A. phagocytophilum *infection is negatively associated with altitude (masl) is in accordance with previous findings [27]. It is also shown that ticks are found at altitudes up to 1100 masl in Central Europe [7]. For farm B the prevalence of seropositive lambs varied from 67% in 2007 to 14% in 2008, indicating considerable variation between years in *A. phagocytophilum *infection.

### Serology

No antigen from a sheep variant of *A. phagocytophilum *was available. The sensitivity of the serology test may have been improved using a more proper antigen than the heterologous horse variant (*E.equi) *of *A.phagocytophilum*. Earlier studies indicate frequent cross-reactions between different variants of *A. phagocytophilum *[28,29]. However, antibody titre to heterologous strains of *Anaplasma *may be lower than to a homologous strain [30] and this might also affect the risk of false negative titres. Unfortunately, titre values were not obtained in the present study.

The time of infection during grazing period is not known and infection may have occurred on spring, summer and/or autumn pastures. It has, however, been shown that antibody titres can persist for at least 6 months in sheep after the primary infection [31,32]. Although different variants may cause different serological responses [17,33] and a spring infection might give reduced titre values in the autumn, it is expected that serology at the age of 137 days is a reliable indicator of infection or no infection if lambs have been infected during the grazing season [5].

### Weight gain

A difference of 1.34 kg between seropositive and seronegative lambs to *A. phagocytophilum *infection is less than reported from a previous study showing 3.8 kg weight difference [13]. Other studies have also shown relatively higher losses to TBF [8,9,13,14]. Still, if the modest presumption that 300 000 [2] lambs are infected by *A. phagocytophilum *each year in Norway, a 1.34 kg weight loss implies a reduction of 165 tons of lamb meat per year. Also, a reduced carcass weight may cause a reduced carcass quality (muscling), grade and lower price per kg.

No significant difference of spring weight between lambs that were seropositive and seronegative to *A. phagocytophilum *infection in autumn was observed. Average age at spring weight recordings vary between 3 - 63 days (mean = 26, S.D. = 13). This together with the fact that *A. phagocytophilum *infection might affect the live weight for several months after infection [14] implies that weight differences are likely to accumulate with increasing age i.e. at weaning weight. Also, lambs that show seroresponse to *A. phagocytophilum *infection in autumn, are not necessarily infected in spring, but possibly later in the grazing period.

It is known that there are several genetic variants of *A. phagocytophilum *and that these cause different clinical signs with varying haematological and serological response [16-18]. A genetic variant of *A. phagocytophilum *(GenBank acc. no. U02521) showed no fever, weight reduction or other signs of clinical illness after experimental inoculation [34]. Different variants of the bacterium may show significantly different clinical reaction and cross-immunity [18]. The variants of *A. phagocytophilum *involved in this study are unknown. The variants involved may partly explain the variation in direct and indirect losses to the *A. phagocytophilum *infections observed. However, additional stress factors as individual condition, management and other infections are also important for the outcome of an infection with *A. phagocytophilum.*

Overall, mean weaning weight and daily weight gain of the lambs in this study population were higher than the county and national average (Table 1). Pasture quality and stress levels in general affect performance and robustness to disease. High quality pastures, shown by average weight gain and autumn live weight above national and county average, and possibly low stress levels may explain a relatively low weight difference between seropositive and seronegative lambs.

The analysis of variance for weaning weight showed that the effect of age at weight recording, age of mother, sex, rearing rank and mother explained much more of the variation in weight gain than the antibody result (*A. phagocytophilum *infection), indicating that infection with *A. phagocytophilum *does not necessarily affect the weight substantially.

### Farm characteristics

The results of this study supports previous findings that ticks and *A. phagocytophilum *infected lambs can be found even if farmers perceive that there are no ticks on their pastures and no observed cases of TBF in their flock [13]. It also indicates that prophylactic treatment with acaricides does not prevent infection, as high seroprevalence of *A. phagocytophilum *was observed in flocks where lambs were treated with acaricides. It is previously shown that lambs treated with acaricides seroconvert after only 3 weeks on tick pasture [5,35]. Routine use of acaricides is not a sustainable measure due to the possibility of developing acaricide resistance [36-38]. The use of acaricides also has practical limitations as regular treatment of free ranging lambs on forest and mountain pastures is not feasible during the grazing season. Use of acaricides has however shown reduced incidence of secondary infections to TBF [37].

The direct losses of lambs on pasture in 2007 and 2008 were in Norway 8.4 and 7.7% respectively. Corresponding losses were 12.0 and 10.4% in the county of Møre and Romsdal [22]. In this study population lamb losses to the predator wolverine (*Gulo gulo*) were documented in two flocks. The actual causes of deaths in general were unknown in this study, which is the general case for most lamb losses during summer pasturing [25,12]. High lamb losses during summer pasturing is a great worry for the sheep industry and TBF is shown to give high losses in some flocks [8]. This study does however not show any correlation between seroprevalence and lamb losses, and the interpretation of TBF as a possible cause of lamb losses in this study is not clear.

## Conclusion

In summary, the present study supports previous findings that *A. phagocytophilum *infection is widespread. It also shows that an *A. phagocytophilum *infection affects live weight. However, *A. phagocytophilum *infections do not always cause substantial direct or indirect losses.

## Competing interests

The authors declare that they have no competing interests.

## Authors' contributions

All authors contributed in designing the study and supervising the writing of the manuscript. LG was responsible for data collection, the statistical analysis and writing the draft manuscript. IO contributed particularly with input on statistical analysis. SS contributed particularly with input into the discussion of the results. All authors approved the final manuscript.
